# 原发免疫性血小板减少症合并弥漫性肺泡出血的临床特征与预后

**DOI:** 10.3760/cma.j.cn121090-20250308-00120

**Published:** 2025-12

**Authors:** 倬玉 安, 海霞 付, 芃 赵, 晓璐 朱, 云 何, 瑾 吴, 辰璁 王, 丽萍 杨, 晓辉 张

**Affiliations:** 北京大学人民医院，北京大学血液病研究所，国家血液系统疾病临床医学研究中心，血液肿瘤细胞和基因治疗北京市重点实验室，北京 100044 Peking University People's Hospital, Peking University Institute of Hematology, National Clinical Research Center for Hematologic Diseases, Beijing Key Laboratory of Cell and Gene Therapy for Hematologic Malignancies, Beijing 100044, China

**Keywords:** 血小板减少, 弥漫性肺泡出血, 临床特征, Thrombocytopenia, Diffuse alveolar hemorrhage, Clinical characteristics

## Abstract

**目的:**

探讨原发免疫性血小板减少症（ITP）合并弥漫性肺泡出血（DAH）的临床特征及预后。

**方法:**

回顾性分析2015年1月至2024年6月北京大学人民医院收治的5例ITP合并DAH患者数据，统计患者的基本特征、临床症状、实验室检查、影像学检查及治疗反应，评估患者的生存情况和预后因素。

**结果:**

患者均为女性，中位年龄56（38～66）岁，4例为慢性ITP，3例合并感染。主要症状为皮肤黏膜出血（3/5）、呼吸困难（3/5）及咯血（2/5），中位PLT 51（1～83）×10^9^/L，D-二聚体显著升高［中位值870（220～8 578）ng/ml］。胸部CT示双肺弥漫性磨玻璃影（3/5）、实变影（1/5）及支气管充气征（1/5）。3例完全缓解，2例死亡。

**结论:**

ITP合并DAH以急性呼吸衰竭为主要表现，D-二聚体升高及感染控制失败是死亡的高危因素，早期联合免疫抑制与抗感染治疗可改善预后。

## Abstract (en)

**Objective:**

This study aimed to investigate the clinical characteristics and prognosis of primary immune thrombocytopenia（ITP）complicated with diffuse alveolar hemorrhage（DAH）.

**Methods:**

This study retrospectively analyzed the clinical data of five patients with ITP complicated with DAH admitted to Peking University People's Hospital from January 2015 to June 2024. Basic characteristics, clinical symptoms, laboratory tests, imaging findings, and treatment responses were summarized, and survival and prognostic factors were evaluated.

**Results:**

All patients were female, with a median age of 56（38–66）years. Four cases were diagnosed with chronic ITP, and three cases were complicated by infection. The main symptoms included mucocutaneous bleeding（3/5）, dyspnea（3/5）, and hemoptysis（2/5）. The median PLT was 51（1–83）×10^9^/L. D-dimer was significantly increased［median 870（220–8 578）ng/ml］. Chest computed tomography revealed diffuse ground-glass opacities in both lungs（3/5）, consolidation（1/5）, and air bronchogram（1/5）. Three patients achieved complete remission, whereas two patients died.

**Conclusion:**

ITP complicated by DAH mainly manifests as acute respiratory failure. Increased D-dimer and failure to control infection are high-risk factors for death. Therefore, early combined immunosuppression and anti-infection treatment improves prognosis.

原发免疫性血小板减少症（primary immune thrombocytopenia, ITP）是由抗血小板自身抗体介导的获得性出血性疾病，其主要病理机制涉及血小板破坏加速与巨核细胞生成血小板受损[1]–[2]。典型临床表现包括皮肤黏膜出血点、紫癜及鼻衄，重症患者可发生颅内出血或内脏出血，是急诊救治的主要指征[3]–[4]。弥漫性肺泡出血（diffuse alveolar hemorrhage, DAH）作为呼吸系统重症，以肺泡毛细血管基底膜完整性破坏为特征，病理表现为红细胞渗入肺泡腔及肺间质[5]–[7]，咯血、贫血、急性呼吸衰竭与双肺弥漫性磨玻璃影为其主要临床表现[5]–[6],[8]。DAH可被视作与原发病相关的伴随症状[9]。系统性红斑狼疮、抗中性粒细胞胞质抗体（antineutrophil cytoplasmic antibodies，ANCA）相关性血管炎等自身免疫性疾病是其常见病因[5]–[6]，感染、心血管疾病与部分药物的使用也可能引起DAH[10]–[13]。

ITP合并DAH的发病机制可能是血小板极度减少（<20×10^9^/L），肺微血管内皮细胞缺乏血小板源性生长因子无法维持结构稳定，免疫复合物沉积激活补体系统，共同加剧肺泡出血风险[14]。ITP合并DAH的病例报告不足10例，提示其属于ITP罕见临床事件。患者表现为突发性呼吸困难（发生率92％）、低氧血症（经皮血氧饱和度<90％）及HGB急剧下降（24 h内下降≥20 g/L），但仅约45％出现典型咯血症状，易与肺炎、急性呼吸窘迫综合征混淆[14]–[16]。诊断需结合支气管肺泡灌洗液（bronchoalveolar lavage fluid，BALF）检测，若灌洗液呈进行性血性，含铁血黄素细胞阳性即可确诊[11]。影像学上，高分辨率CT（high-resolution computed tomography，HRCT）多显示双肺弥漫性磨玻璃影，分布以下肺野为主，部分病例可见支气管充气征或小叶间隔增厚[6]。

现有研究多局限于个案报道，缺乏大样本队列数据，ITP合并DAH无标准治疗方案。本文通过回顾性分析ITP合并DAH患者的临床特征及预后，揭示其临床异质性，为临床诊疗提供参考。

## 病例与方法

1. 病例：本研究为单中心回顾性队列研究，纳入2015年1月至2024年6月北京大学人民医院收治的ITP合并DAH患者。电子病历系统（EMRS V5.0）筛选检索的关键词包括“免疫性血小板减少症”、“肺泡出血”、“DAH”及相关国际疾病分类第10版（ICD-10）编码（D69.3、J84.8）。ITP的诊断遵循《成人原发免疫性血小板减少症诊断与治疗中国指南（2020年版）》[17]，要求PLT<100×10⁹/L且排除药物、感染、自身免疫病等继发因素。DAH的确诊需满足以下标准中至少2项：①BALF呈进行性血性改变且含铁血黄素细胞≥5％；②HRCT显示双肺新发弥漫性磨玻璃影或实变影；③HGB 24 h内下降≥20 g/L且无其他明确出血部位[6]。排除合并系统性红斑狼疮、ANCA相关性血管炎、抗磷脂综合征等已知DAH病因，以及近期（<4周）接受肺毒性药物治疗或临床资料不完整的病例，最终纳入5例患者进行分析。

2. 数据采集与评估方法：数据通过结构化电子病历系统提取，涵盖基本特征（年龄、性别、ITP病程分型）、临床症状及体征（皮肤黏膜出血、咯血、氧合指数、是否有机械通气需求）、实验室检查（PLT、网织血小板比例、D-二聚体）及影像学检查资料。病原学检测采用BALF培养、PCR及宏基因组二代测序。

3. 疗效和随访：完全缓解定义为症状消失、PLT≥50×10⁹/L持续7 d且影像学病灶吸收≥75％；部分缓解定义为症状改善、PLT≥30×10⁹/L伴病灶吸收≥50％；治疗失败定义为死亡或需体外膜肺氧合支持[6]。随访方式为查阅电子病历系统及电话随访，随访截止日期为2024年6月30日。

4. 统计学处理：数据分析采用SPSS 26.0软件，连续变量以中位数（范围）表示，分类变量以频数（百分比）描述。

## 结果

1. 基线临床特征：本研究共纳入5例ITP合并DAH患者，均为女性，中位年龄56（38～66）岁。4例患者ITP病程>12个月（慢性型），1例为新诊断型（病程<3个月）。合并基础疾病包括糖尿病（1例）、高血压（1例）、桥本甲状腺炎（1例）及恶性肿瘤（乳腺癌1例）。3例合并感染，其中真菌感染（黄曲霉/烟曲霉）2例，病毒性肺炎（新型冠状病毒/巨细胞病毒）2例，细菌感染（鲍曼不动杆菌）2例（表1）。

**表1:** 5例原发免疫性血小板减少症（ITP）合并弥漫性肺泡出血（DAH）患者的基线临床特征

例号	DAH发病年龄	ITP病程分型	基础疾病	合并感染	DAH起病形式	主要临床症状	DAH发病前治疗史
1	56岁	慢性	无	巨细胞病毒、黄曲霉混合感染	急性	皮肤黏膜出血、咯血、呼吸困难	泼尼松10 mg/d+环孢素75 mg每日2次（发病前6个月）
2	58岁	慢性	糖尿病、高血压、乳腺癌	无	亚急性	皮肤黏膜出血	甲泼尼龙80 mg/d×14 d（发病前3个月），后调整为泼尼松15 mg/d维持
3	66岁	新诊断	无	病毒性肺炎、烟曲霉合并鲍曼不动杆菌	急性	呼吸困难	无免疫抑制治疗史，曾短期使用艾曲泊帕50 mg/d（发病前2周停药）
4	48岁	慢性	无	新型冠状病毒合并鲍曼不动杆菌	亚急性	皮肤黏膜出血	利妥昔单抗（375 mg/m^2^/周×4周，发病前4个月）+泼尼松5 mg/d维持
5	38岁	慢性	桥本甲状腺炎	无	急性	咯血、呼吸困难	泼尼松20 mg/d+环孢素50 mg每日2次（发病前6个月）

2. DAH发病情况：患者的DAH起病形式以急性为主（3/5），从ITP确诊至DAH发生的中位间隔时间为8（1～34）年。患者的主要临床症状包括皮肤黏膜出血（3/5）、咯血（2/5）及呼吸困难（3/5）。实验室检查显示，发病时中位PLT为51（1～83）×10^9^/L，3例PLT<30×10^9^/L。D-二聚体水平显著升高［中位值870（220～8 578）ng/ml］，其中1例死亡病例的D-二聚体值最高（8 578 ng/ml）。所有患者均存在低氧血症［氧分压中位值51（45～85）mmHg］（1 mmHg＝0.133 kPa），3例合并代谢性酸中毒（pH<7.40）。患者DAH发病前治疗情况显示，4例患者近6个月内接受过中高强度免疫抑制治疗，包括糖皮质激素联合环孢素方案（2例）、大剂量甲泼尼龙冲击治疗（1例）和利妥昔单抗治疗（1例），仅1例新诊断ITP患者未接受免疫抑制治疗。3例患者在DAH诊断前已存在感染：例1在DAH确诊前2周诊断巨细胞病毒血症，例3在DAH确诊前5 d存在病毒性肺炎表现，例4在新型冠状病毒感染后10 d出现DAH症状。

3. 实验室及影像学检查：BALF分析显示，4例呈血性或淡血性外观，3例含铁血黄素细胞阳性。胸部CT均表现为双肺弥漫性病变，其中3例表现为磨玻璃影，1例表现为实变影，1例表现为支气管充气征。

4. 治疗情况：所有患者均接受大剂量糖皮质激素治疗，甲泼尼龙剂量范围为24～500 mg/d，疗程3～7 d。3例联合免疫抑制剂治疗，包括环孢素（50 mg每日2次）、吗替麦考酚酯（0.75 g每日2次）及利妥昔单抗（375 mg/m^2^单次输注）。呼吸支持方式包括低流量吸氧（2例）、高流量氧疗（1例）及有创机械通气（2例）。3例达到完全缓解（症状及影像学异常消失），中位缓解时间为7（3～21）d。2例死亡，合并多重耐药菌感染（表2）。

**表2:** 5例原发免疫性血小板减少症合并弥漫性肺泡出血患者的治疗情况与预后

例号	免疫治疗方案	呼吸支持方式	合并感染	转归
1	甲泼尼龙24 mg/d×7 d+环孢素50 mg每日2次	有创机械通气	巨细胞病毒、黄曲霉混合感染	死亡
2	甲泼尼龙120 mg/d×5 d+吗替麦考酚酯0.75 g每日2次	高流量氧疗	无	完全缓解
3	甲泼尼龙500 mg/d×3 d	有创机械通气	烟曲霉合并鲍曼不动杆菌	死亡
4	甲泼尼龙500 mg/d×5 d+利妥昔单抗375 mg/m^2^	低流量吸氧	新型冠状病毒合并鲍曼不动杆菌	完全缓解
5	甲泼尼龙320 mg/d×7 d	低流量吸氧	无	完全缓解

5. 预后：中位随访29（20～607）d，2例患者死亡，3例完全缓解（表2）。死亡病例存在以下特征：①多重耐药菌感染；②D-二聚体>1 500 ng/ml；③需有创机械通气。完全缓解病例中2例（2/3）未合并感染，且起病时该2例患者PLT>30×10^9^/L。

## 讨论

ITP合并DAH是一种罕见的临床事件，目前仅有少量病例报道，其临床特征和治疗反应仍未得到充分研究[14]–[16]。本研究纳入5例ITP合并DAH患者，旨在为DAH的早期识别、治疗策略和预后评估提供一定的依据。

本研究中的患者均为女性，且年龄范围较广（38～66岁）。ITP的病程大多为慢性型（4/5），合并基础疾病包括糖尿病、高血压及恶性肿瘤等。所有患者均存在血小板减少（PLT<100×10^9^/L），其中3例PLT<30×10^9^/L。D-二聚体水平升高是明显的特征，死亡病例的D-二聚体水平显著高于存活患者，提示D-二聚体作为DAH的潜在标志物，可能反映了肺微血管内皮细胞的损伤程度[18]。同时，低氧血症和代谢性酸中毒是常见的临床表现，提示肺泡出血可能导致呼吸衰竭[19]–[20]。

从临床症状来看，皮肤黏膜出血、咯血及呼吸困难是ITP合并DAH的常见表现，而呼吸困难的发生比例为3/5。咯血虽然是DAH的典型症状，但在本研究中仅2例表现为咯血，提示咯血可能并非ITP合并DAH患者的必然症状，且易与肺炎、急性呼吸窘迫综合征等混淆[21]–[25]。因此，对于出现突发性呼吸困难或血氧下降的ITP患者，应及时考虑并排除DAH可能。本研究中2例患者存在病毒感染（新型冠状病毒/巨细胞病毒），提示病毒感染可能是ITP患者发生DAH的重要诱因。最新研究表明，病毒感染可通过多种机制促进DAH发生，包括直接损伤肺泡上皮细胞和毛细血管内皮细胞[13]；触发细胞因子风暴，导致肺泡-毛细血管屏障功能受损[26]；激活补体系统，加剧免疫复合物介导的肺损伤[27]。Löffler等[10]报道，2例免疫抑制患者在新型冠状病毒感染后发生DAH，与本研究的结果一致。在ITP患者中，长期免疫抑制治疗可能导致免疫监视功能受损，使患者更易受病毒感染影响，增加DAH发生风险。这一发现提示，对于接受免疫抑制治疗的ITP患者，应加强病毒感染监测和预防。

胸部CT是诊断ITP合并DAH的重要工具[28]，本研究中所有患者均表现为双肺弥漫性病变，其中磨玻璃影为最常见的影像学特征（3/5）。此外，部分患者也表现为实变影和支气管充气征。BALF的分析结果显示，血性或淡血性外观与含铁血黄素细胞阳性是DAH的典型表现[29]，进一步支持了ITP合并DAH的诊断[24]。

本研究患者均接受了大剂量糖皮质激素治疗，且大多数患者接受了免疫抑制剂联合治疗。3例患者联合使用环孢素、吗替麦考酚酯或利妥昔单抗，并取得了较好的治疗效果。现有文献也支持联合应用多种免疫抑制剂治疗DAH的有效性[14],[30]。呼吸支持方面，部分患者需要机械通气，且大多数患者最终获得缓解，表明早期识别并及时干预可以有效改善患者的预后[31]。既往研究揭示，机械通气与DAH的不良预后结局相关[32]–[33]，本研究中，接受机械通气的患者同样预后较差，提示需要机械通气治疗可能是ITP合并DAH患者预后不良的预测因素。2例死亡患者均合并多重耐药菌感染，且需要有创机械通气，提示在免疫抑制治疗过程中，感染的控制至关重要。既往文献报道，重组人活化因子Ⅶ（recombinant activated factor Ⅶ，rFⅦa）在ITP和DAH中均成功应用[27],[34]。rFⅦa可促进局部凝血酶形成，加速止血过程，尤其适用于常规治疗无效的危重患者。鉴于ITP合并DAH的高病死率，对于常规免疫抑制和抗感染治疗效果不佳的患者，早期应用rFⅦa等止血药物是潜在的治疗选择。

本研究样本量较小，研究结果的普遍性和适用性仍需进一步验证。未来研究应扩大样本量，探索ITP合并DAH的分子机制、早期生物标志物以及个体化治疗方案。此外，考虑到多重耐药菌感染对预后的重要影响，未来应加强免疫抑制治疗期间的抗感染管理，并探索更为精准的抗感染策略。
